# Identification of *CCSER1* missense variants and diplotypes as molecular markers for growth and lambing-related traits in Junken meat sheep

**DOI:** 10.3389/fvets.2026.1922937

**Published:** 2026-09-17

**Authors:** Sanchuan Zhao, Binpeng Xi, Qian Yu, Wenzhe Zhang, Yan Chen, Junlin Lan, Zongsheng Zhao, Hua Yang

**Affiliations:** 1 College of Animal Science and Technology, Shihezi University, Shihezi, China; 2 State Key Laboratory of Sheep Genetic Improvement and Healthy Production, Xinjiang Academy of Agricultural and Reclamation Science, Shihezi, China; 3 Key Laboratory of Animal Genetics and Breeding on the Tibetan Plateau, Ministry of Agriculture and Rural Affairs, Lanzhou Institute of Husbandry and Pharmaceutical Sciences, Chinese Academy of Agricultural Sciences, Lanzhou, China

**Keywords:** association analysis, coiled-coil serine rich protein 1 (CCSER1) gene, growth traits, Junken meat sheep, lambing-related traits

## Abstract

To validate the potential role of *CCSER1*, a candidate gene for litter size and yearling weight identified by selection signature analysis, in lambing performance and growth development of Junken meat sheep, *CCSER1* polymorphisms were genotyped using the Sequenom MassARRAY^®^ SNP assay. Association analysis was conducted to assess the associations of *CCSER1* polymorphisms and SNP2–SNP3 diplotypes with growth and lambing-related traits, and reverse transcription quantitative PCR (RT-qPCR) was used to determine the expression profile of *CCSER1* in the ewes' digestive, developmental and reproductive tissues. The results showed three missense mutations in the *CCSER1* gene of Junken meat sheep were identified, including SNP1 (XM_042251242.2: c.2557C>T) in exon 11, SNP2 (XM_042251242.2: c.1206A>G) and SNP3 (XM_042251242.2: c.16T>C) in exon 2. Three SNPs were exploratorily predicted to alter the secondary and tertiary structures of the *CCSER1* protein. A single-stage mixed model was applied to evaluate the associations between individual SNP genotypes and phenotypic variation, with Bonferroni correction subsequently performed for multiple testing within each trait. The results showed that the associations of SNP2 with ABW (adjusted *P* = 0.0024) and SNP3 with YW (adjusted *P* = 0.022) and ADG2 (adjusted *P* = 0.039) remained statistically significant after Bonferroni correction. Phasing analysis of SNP2 and SNP3 identified four chromosome-level haplotypes and six corresponding diplotypes. A significant diplotype association was observed for YW (adjusted *P* = 0.039). Tukey's HSD test indicated that individuals carrying the T-A/T-A or T-A/T-G diplotypes exhibited significantly higher YW compared with those carryingT-G/T-G diplotype. The RT-qPCR analysis revealed a tissue-specific expression profile of *CCSER1*, with the highest expression detected in the abomasum (*P* < 0.01). In addition, *CCSER1* expression levels in the lung and ovary were significantly higher than those in the liver, kidney, skin, brain, and pituitary gland (*P* < 0.01). These findings suggest that *CCSER1* is a candidate gene for growth and lambing-related traits in Junken meat sheep, and that SNP2, SNP3, T-A/T-A and T-A/T-G may serve as useful molecular markers for marker-assisted selection to improve lambing performance and growth development in sheep.

## Introduction

Sheep farming is an important source of global meat production, and lambing performance and growth rate are core economic traits that directly affect the profitability of sheep production (1). In recent years, with the rapid development of modern intensive farming and the continuous advancement of precision breeding, the development of new meat sheep breeds combining high fecundity with greater body weight has become an important direction in modern animal breeding (2). However, litter size and body weight often show a negative correlation, as multiple births are frequently accompanied by lower lamb birth weight and slower growth and development (3). Therefore, identifying key genetic markers that can simultaneously contribute to increased prolificacy and greater body weight has become a major focus in sheep breeding.

In our previous studies, genome-wide association study (GWAS) and selection signature analysis identified the coiled-coil serine-rich protein 1 gene (*CCSER1*) as a candidate gene associated with litter size (4) and yearling weight (5) in sheep. The *CCSER1* gene is located on chromosome 6 in sheep, consists of 11 exons, and encodes 899 amino acids (6). Cell biological studies have shown that the protein encoded by *CCSER1* is mainly localized to key structures such as the midbody during late mitotic cytokinesis and the γ-tubulin ring complex, and serves as an important structural or regulatory component required for normal cell division and the maintenance of chromosomal and genomic stability (7, 8). This fundamental role in cell proliferation regulation may confer pleiotropic potential on *CCSER1* in economically important traits of livestock. Previous studies have shown that *CCSER1* is not only significantly associated with diverse phenotypes, including coat characteristics in Northwest Tibetan cashmere goats (9), disease resistance in Merino sheep (10), and milk yield in Yuansheng Aite dairy sheep (11), but also represents a key gene involved in the regulation of growth and reproductive performance in sheep. Liu et al. (12) identified *CCSER1* as an important selected gene related to reproduction and development in the Xiangdong black goat population. Li et al. (13) confirmed, using GWAS, that this gene has a significant genetic effect on body weight traits in Hu sheep from 3 to 7 months old. Although multiple studies have demonstrated the important role of *CCSER1* in reproduction, growth, and development in sheep and goats, whether the genetic polymorphisms exist in *CCSER1* gene of Junken meat sheep, a newly developed prolific meat sheep breed (14), and how these polymorphisms regulate growth and ewe reproductive traits remain unclear.

Therefore, in this study, candidate gene analysis and bioinformatics approaches were used to identify the mutation sites in the *CCSER1* gene and predict the effects of SNPs on changes in *CCSER1* protein structure. *CCSER1* polymorphisms were genotyped using the Sequenom MassARRAY^®^ SNP assay. Genotype-phenotype association analyses were performed to evaluate the effects of *CCSER1* SNPs on growth and lambing-related traits. In addition, SNP2 and SNP3 within the same LD block were phased to reconstruct chromosome-level haplotypes and individual diplotypes, followed by diplotype-phenotype association analysis. In addition, reverse transcription quantitative PCR (RT-qPCR) was used to analyze the transcriptional profile of *CCSER1* in digestive, developmental, and reproductive organs of ewes. This study aimed to evaluate population-specific associations between *CCSER1* variants and economically relevant traits. These results may provide a basis for the application of marker-assisted selection in improving growth and lambing-related traits in sheep.

## Materials and methods

### Animal ethics statement

This study was carried out in accordance with the Guidelines for the Care and Use of Laboratory Animals and approved by the Experimental Animal Care and Use Committee of Xinjiang Academy of Agricultural and Reclamation Sciences (Shihezi, China, ethic committee approval number: XJNKKXY-2020–34, 30 December 2020).

### Sample collection

A total of 265 healthy Junken mutton sheep aged 3–6 years were selected from the Sheep Breeding Farm of Xinjiang Academy of Agricultural and Reclamation Science, comprising 235 ewes and 30 rams. All individuals were born and raised within the original breeding population without introgression from other sheep breeds. The animals were maintained under the same environmental and management conditions and had free access to feed. Before sample collection, all sheep were fasted for 12 h with free access to water to ensure sampling standardization and data accuracy. Blood samples were collected from the jugular vein into EDTA anticoagulant tubes, gently inverted to ensure complete mixing, and stored at −20 °C until genomic DNA extraction.

Three 1-year-old female Junken meat sheep with similar body weights and same genotypes of SNP1, SNP2, and SNP3 were sampled, and tissues were collected following standardized procedures. Briefly, anesthesia was induced by sequential intramuscular administration of diazepam (0.5–1.0 mg/kg; Gansu Ankang Pharmaceutical Co., Ltd., China) and scopolamine (0.01–0.04 mg/kg; Kaimei Gene, USA), followed by intravenous administration of thiopental sodium (10–20 mg/kg; Shanghai Xinya Pharmaceutical Co., Ltd., China). After anesthesia, the sheep were euthanized by jugular exsanguination, and tissue samples were immediately collected, including the heart, liver, lung, kidney, skin, skeletal muscle, brain, pituitary gland, rumen, abomasum, duodenum, ileum, uterus, and ovary. The collected tissues were rapidly transferred into RNase-free cryopreservation tubes and immediately immersed in liquid nitrogen to prevent RNA degradation. These samples were subsequently used for RNA extraction.

### Phenotypic data processing

Phenotypic data of the experimental sheep population were collected and processed, including the litter size of each parity (LS) and lamb birth weight of ewes from 2020 to 2024, as well as the birth weight (BW), weaning weight at 3 months old (WW) and yearling weight (YW) in the experimental population. Microsoft Excel 2019 was used to calculate the average birth weight per litter (ABW), average daily gain from birth to weaning (ADG1), average daily gain from birth to 12 months old (ADG2) and average daily gain from weaning to 12 months old (ADG3) for each sheep. The calculation formulas were as follows. To ensure the reliability of subsequent association analyses, phenotypic records deviating from the mean by more than three standard deviations were excluded (15).


$$\begin{array}{l} ABW_{i}=\frac{\sum_{k=1}^{m_{i}}W_{ik}}{m_{i}} \end{array}$$

Where *ABW*_*i*_ is the average lamb birth weight of the ewe at the *i*-th parity; *m*_*i*_ is the number of lambs born to the ewe at the *i*-th parity; *W*_*ik*_ is the actual birth weight of the *k*-th lamb in the *i*-th parity.


$$\begin{array}{l} ADG1=\frac{\left(WW-BW\right)\times 1000}{90}\\ ADG2=\frac{\left(YW-BW\right)\times 1000}{365}\\ ADG3=\frac{\left(YW-WW\right)\times 1000}{275} \end{array}$$

Where *ADG*1 is the average daily gain of each sheep from birth to weaning; *ADG*2 is the average daily gain from birth to 12 months old; and *ADG*3 is the average daily gain from weaning to 12 months old. *BW*, *WW*, and *YW* denote birth weight, weaning weight and yearling weight, respectively.

### DNA isolation and genotyping

Genomic DNA was extracted from blood samples using the RelaxGene Blood DNA System (TIANGEN Biotech Co., Ltd., Beijing, China). DNA quality was assessed by 1.0% agarose gel electrophoresis, and DNA concentration was measured using a NanoDrop™ 2000 spectrophotometer (Thermo Fisher Scientific, Waltham, MA, USA). Samples with DNA concentrations greater than 20 ng/μL and OD260/OD280 ratios ranging from 1.7 to 1.9 were considered suitable for subsequent experiments.

Single-base extension primers for the three target loci were designed using Assay Design software (version 3.1) (Sequenom, Inc., San Diego, CA, USA) based on the ovine *CCSER1* reference transcript XM_042251242.2 annotated on the ARS-UI_Ramb_v3.0 reference genome assembly. The primer information is listed in Table 1, and all primers were synthesized by GENWIZ Biotechnology Co., Ltd. (Tianjin, China). Subsequently, the target SNP loci in the experimental population were genotyped using the Sequenom MassARRAY^®^ SNP genotyping platform. The experimental procedure included PCR amplification, shrimp alkaline phosphatase (SAP) treatment, single-base extension reaction, mass spectrometric detection, and genotype data acquisition.

**Table 1:** Primers sequence information of the Sequenom MassARRAY^®^ SNP genotyping.

SNPs	Primer sequences (5^′^-3^′^)
SNP1	F: ACGTTGGATGGTCCAAACCTTCCTGTAAAG
	R: ACGTTGGATGGAACTCTATGAAGCCAGCAG
SNP2	F: ACGTTGGATGAAGCTCCCCAAGGAAGTTTG
	R: ACGTTGGATGTGATGATCACCAGAAGAGGG
SNP3	F: ACGTTGGATGTAGCTATCACAGCACAAGCC
	R: ACGTTGGATGTGAATATTGGCAACCGGGAG

### Function prediction

The wild-type and mutant sequences of the *CCSER1* gene were retrieved from the Ensembl database (https://www.ensembl.org/index.html; accessed on 7 May 2026). The secondary and tertiary structures of the target protein before and after missense mutations were predicted using SOPMA (https://npsa.lyon.inserm.fr/cgi-bin/npsa_automat.pl?page=/NPSA/npsa_sopma.html; accessed on 7 May 2026) and SWISS-MODEL (https://swissmodel.expasy.org/; accessed on 7 May 2026), respectively. All parameters were set to default values.

To elucidate the biological function of the protein encoded by the candidate gene *CCSER1* and identify its potential interacting molecules, a protein-protein interaction (PPI) network was constructed using the Search Tool for the Retrieval of Interacting Genes/Proteins database, STRING v12.0 (https://string-db.org/; accessed on 8 May 2026), with *Ovis aries* selected as the reference organism.

### Population genetic analysis

R software (version 4.5.3) (R Foundation for Statistical Computing, Vienna, Austria) was used to calculate the genotype frequency, allele frequency, minor allele frequency (MAF), observed heterozygosity (Ho), expected heterozygosity (He), effective number of alleles (Ne), and polymorphism information content (PIC) for each SNP locus. Linkage disequilibrium (LD) analysis and Hardy-Weinberg equilibrium (HWE) tests of the SNP loci were performed using Haploview software (version 4.2) (16).

### Association analysis of SNP loci and phenotypic traits

All SNP–phenotype associations were analyzed using single-stage models in JMP Pro software (version 18.0.0), with genotype and the relevant non-genetic systematic effects fitted simultaneously.

For parity-specific litter size (LS), a linear mixed model was fitted as:


$$\begin{array}{l} y_{ijkl}=\mu +G_{i}+Y_{j}+P_{k}+u_{l}+e_{ijkl} \end{array}$$

where *y*_*ijkl*_ is the litter-size record; μ is the overall mean; *G*_*i*_ is the fixed effect of the *i*-th genotype; *Y*_*j*_ is the fixed effect of the *j*-th lambing year; *P*_*k*_ is the fixed effect of the *k*-th parity; *u*_*l*_ is the random effect of the *l*-th ewe, included to account for repeated records within the same individual; *e*_*ijkl*_ and is the random residual error.

For average birth weight per litter (ABW), the following linear mixed model was fitted:


$$\begin{array}{l} y_{ijklm}=\mu +G_{i}+Y_{j}+P_{k}+B_{l}+u_{m}+e_{ijklm} \end{array}$$

where *y*_*ijklm*_ is the average birth weight per litter; μ is the overall mean; *G*_*i*_ is the fixed effect of genotype; *Y*_*j*_ is the fixed effect of lambing year; *P*_*k*_ is the fixed effect of parity; *B*_*l*_ is the fixed effect of litter type; *u*_*m*_ is the random effect of ewe; *e*_*ijklm*_ and is the random residual error.

For birth weight (BW), weaning weight (WW), and yearling weight (YW), general linear models were fitted as:


$$\begin{array}{l} y_{ijklmn}=\mu +G_{i}+Y_{j}+B_{k}+P_{l}+S_{m}+e_{ijklmn} \end{array}$$

where *y*_*ijklmn*_ represents BW, WW, or YW; μ is the overall mean; *G*_*i*_ is the fixed effect of genotype; *Y*_*j*_ is the fixed effect of birth year; *B*_*k*_ is the fixed effect of birth type; *P*_*l*_ is the fixed effect of maternal parity; *S*_*m*_ is the fixed effect of sex; *e*_*ijklmn*_ and is the random residual error.

For ADG1 and ADG2, the corresponding initial body weight was additionally included as a continuous covariate:


$$\begin{array}{l} y_{ijklmn}=\mu +G_{i}+Y_{j}+B_{k}+P_{l}+S_{m}+\beta BW_{ijklmn}+e_{ijklmn} \end{array}$$

where *y*_*ijklmn*_ represents ADG1 or ADG2; μ is the overall mean; *G*_*i*_ is the fixed effect of genotype; *Y*_*j*_ is the fixed effect of birth year; *B*_*k*_ is the fixed effect of birth type; *P*_*l*_ is the fixed effect of maternal parity; *S*_*m*_ is the fixed effect of sex; *BW*_*ijklmn*_ is birth weight used as a continuous covariate, and β is the corresponding regression coefficient; *e*_*ijklmn*_ and is the random residual error.

For ADG3, weaning weight was included as the initial-body-weight covariate:


$$\begin{array}{l} y_{ijklmn}=\mu +G_{i}+Y_{j}+B_{k}+P_{l}+S_{m}+\beta WW_{ijklmn}+e_{ijklmn} \end{array}$$

where *y*_*ijklmn*_ represents ADG3; μ is the overall mean; *G*_*i*_ is the fixed effect of genotype; *Y*_*j*_ is the fixed effect of birth year; *B*_*k*_ is the fixed effect of birth type; *P*_*l*_ is the fixed effect of maternal parity; *S*_*m*_ is the fixed effect of sex; *WW*_*ijklmn*_ is weaning weight used as a continuous covariate, and β is its regression coefficient; *e*_*ijklmn*_ and is the random residual error.

To account for multiple testing, Bonferroni correction was applied separately for each phenotypic trait across the three SNP tests. Associations with Bonferroni-adjusted *P* values < 0.05 were considered statistically significant. For associations that remained significant after Bonferroni correction, pairwise comparisons among genotypes were performed using Tukey's HSD test.

### Haplotype reconstruction and diplotype association analysis

Linkage disequilibrium (LD) analysis was first used to identify linked *CCSER1* variants. SNP2 and SNP3, which were located within the same LD block, were subsequently phased using Beagle software (version 5.5). For each individual, phased alleles at SNP2 and SNP3 located on the same chromosome were combined to define chromosome-level haplotypes. The two phased haplotypes carried by each individual were then paired to define the corresponding diplotype. Haplotype frequencies were calculated using the total number of phased chromosomes (2N), whereas diplotype frequencies were calculated using the number of individuals (N). Diplotypes with frequencies below 5% were excluded from phenotype association analyses.

Diplotype–phenotype associations were analyzed in JMP Pro software (version 18.0.0) using the same single-stage statistical models described above for the SNP genotype analyses, with diplotype replacing genotype as the fixed genetic effect. For LS and ABW, ewe identity was included as a random effect to account for repeated records. For BW, WW, YW, ADG1, ADG2, and ADG3, the same systematic fixed effects and continuous covariates used in the corresponding SNP models were retained. Adjusted least-squares means and standard errors were obtained directly from these models.

The *P* values derived from diplotype–phenotype association analyses were subjected to separate multiple-testing correction using the Bonferroni method. Associations with an adjusted (Bonferroni-adjusted *P*-value < 0.05) were considered statistically significant. For associations remaining significant after Bonferroni correction, pairwise comparisons among genotypes were performed using Tukey's HSD test.

### Total RNA extraction and cDNA synthesis

Total RNA was extracted using an RNA/protein extraction kit (Beyotime Biotechnology, Shanghai, China). RNA concentration was measured using a NanoDrop™ 2000 spectrophotometer (Thermo Fisher Scientific, MA, USA), and RNA integrity was assessed by 1% agarose gel electrophoresis. First-strand cDNA was synthesized from total RNA using the EasyScript One-Step gDNA Removal and cDNA Synthesis SuperMix kit (TransGen Biotech, Beijing, China) and stored at −20 °C until further use.

### Detection of *CCSER1* mRNA expression by qPCR

*GAPDH* was used as the internal reference gene, and quantitative real-time PCR (qPCR) was performed to determine the expression profile of *CCSER1* in 14 tissues of Junken meat sheep. The relative expression level of *CCSER1* was calculated using the 2^−Δ*ΔCt*^ method (17). Three biological replicates were included for each sample.

Based on the ovine *CCSER1* and *GAPDH* reference transcript sequences (XM_042251242.2 and NM_001190390.1, respectively), primers were designed using the NCBI Primer-BLAST tool. All primers were synthesized by Sangon Biotech Co., Ltd. (Shanghai, China), and the primer information is shown in Table 2. The qPCR reaction was performed using LightCycler 480 SYBR Green I Master mix (Roche Diagnostics GmbH, Mannheim, Germany) strictly according to the manufacturer's instructions. qPCR detection was conducted using a LineGene 9600 Plus FQD-96A real-time PCR system (Bioer Technology, Hangzhou, China).

**Table 2:** Sequence information of qPCR amplification primers.

Gene name	Primer sequences (5^′^-3^′^)	Length/bp
*CCSER1*	F: ACTCTCGTCCCGCAGGTTA	108
	R: ATATTGGCAACCGGGAGACC	
*GAPDH*	F: CTGACCTGCCGCCTGGAGAAA	149
	R: GTAGAAGAGTGAGTGTCGCTGTT	

## Results

### Analysis of SNP loci in the *CCSER1* gene

In this study, nonsynonymous mutations in the coding region of the *CCSER1* gene were analyzed, and three missense variants were identified. According to the ARS-UI_Ramb_v3.0 reference genome, SNP1 was located at Chr6:33,699,675 in exon 11 with a genomic REF>ALT change of G>A. Relative to the reference transcript XM_042251242.2, this variant was annotated as c.2557C>T and p.Arg853Trp. SNP2 was located at Chr6:35,012,842 in exon 2 with a genomic T>C substitution and was annotated as c.1206A>G and p.Ile402Met. SNP3 was located at Chr6:35,014,032 in exon 2 with a genomic A>G substitution and was annotated as c.16T>C and p.Ser6Pro. Because *CCSER1* is located on the reverse strand, the genomic REF>ALT alleles are complementary to the corresponding transcript-level HGVS nucleotide changes (Figure 1; Table 3).

**Figure 1:**
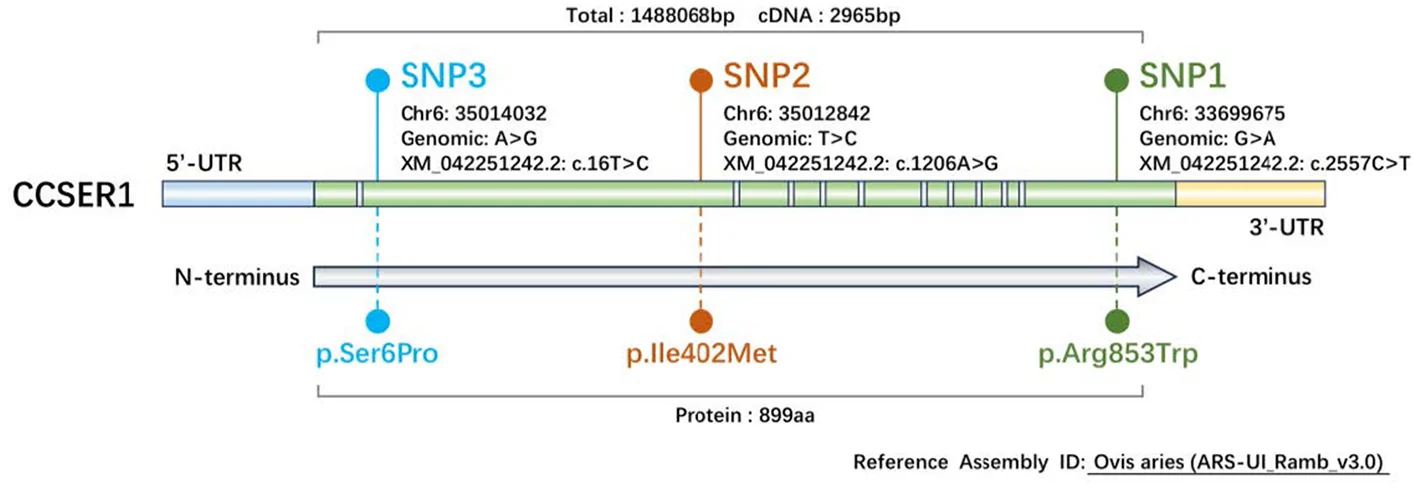
Genetic information of three SNPs in the ovine CCSER1 gene. Blue boxes represent the 5' untranslated region, green boxes represent exons, yellow boxes represent the 3' untranslated region, and gray arrows indicate the orientation of the encoded CCSER1 protein sequence from the N-terminus to the C-terminus. This figure illustrates the genomic structure of the CCSER1 gene, the encoded protein sequence, and the positions of the three SNP loci with their corresponding amino acid variants. The full-length genomic sequence of CCSER1 is 1,488,068 bp, the cDNA sequence is 2,965 bp, and the encoded protein consists of 899 amino acids. Genomic information was obtained from the sheep reference genome assembly ARS-UI_Ramb_v3.0. Because CCSER1 is located on the reverse strand, the gene structure is presented according to its 5'-to-3' transcriptional orientation, with genomic coordinates decreasing from left to right.

**Table 3:** Detailed genomic and protein-level annotation of three SNPs in the *CCSER1* gene.

Variant	Chr	Position	Genomic REF>ALT	Strand	Transcript	HGVS c.	HGVS p.	Exon
SNP1	6	33699675	G>A	reverse	XM_042251242.2	c.2557C>T	p.Arg853Trp	11
SNP2	6	35012842	T>C	Reverse	XM_042251242.2	c.1206A>G	p.Ile402Met	2
SNP3	6	35014032	A>G	reverse	XM_042251242.2	c.16T>C	p.Ser6Pro	2

Genomic coordinates and REF/ALT alleles are reported according to the forward strand of the ARS-UI_Ramb_v3.0 reference genome. Because *CCSER1* is located on the reverse strand, the transcript-level HGVS nucleotide changes are complementary to the corresponding forward-strand genomic REF/ALT alleles and are reported relative to the reference transcript XM_042251242.2. XM_042251242.2 corresponds to the Ensembl transcript ENSOART00285072055.1 used for exon annotation.

Blue boxes represent the 5′ untranslated region, green boxes represent exons, yellow boxes represent the 3′ untranslated region, and gray arrows indicate the orientation of the encoded *CCSER1* protein sequence from the N-terminus to the C-terminus. This figure illustrates the genomic structure of the *CCSER1* gene, the encoded protein sequence, and the positions of the three SNP loci with their corresponding amino acid variants. The full-length genomic sequence of *CCSER1* is 1,488,068 bp, the cDNA sequence is 2,965 bp, and the encoded protein consists of 899 amino acids. Genomic information was obtained from the sheep reference genome assembly ARS-UI_Ramb_v3.0. Because *CCSER1* is located on the reverse strand, the gene structure is presented according to its 5′-to-3′ transcriptional orientation, with genomic coordinates decreasing from left to right.

### Predicted effects of missense mutations on *CCSER1* protein structure and PPI analysis

For the three missense mutation loci, the secondary and tertiary structures of the *CCSER1* protein before and after mutation were visually predicted using SOPMA and SWISS-MODEL. The results showed that the protein encoded by the *CCSER1* gene was mainly composed of three types of secondary structures, namely random coil, α-helix and extended strand (Figures 2A, B; Table 4). The SNP1 mutation decreased the proportions of α-helix and extended strand by 0.44% and 0.78%, respectively, while increasing the proportion of random coil by 1.22% (Figure 2C). The SNP2 mutation reduced the proportion of extended strand by 0.78% (Figure 2D), which was consistent with the predicted effect of the SNP1 mutation. In addition, the SNP2 mutation increased the proportions of α-helix and random coil by 0.67% and 0.11%, respectively (Figure 2D). The SNP3 mutation decreased the proportion of extended strand by 0.56% and increased the proportion of random coil by 0.56%, but did not affect the proportion of α-helix (Figure 2E). Collectively, all three mutations reduced the proportion of extended strand, which generally contributes to protein rigidity and structural stability, and increased the proportion of random coil, a structure characterized by high flexibility and disorder.

**Figure 2:**
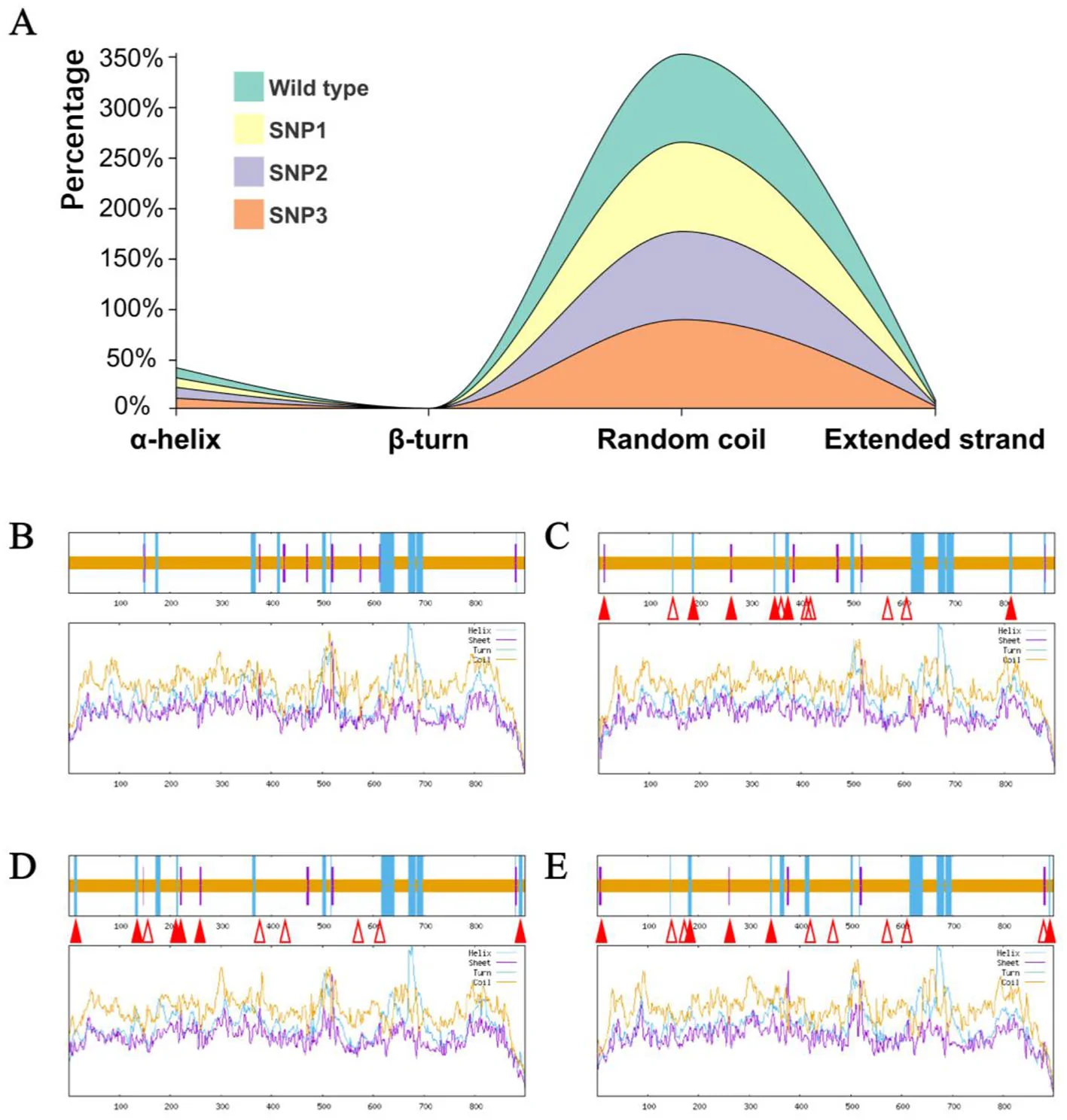
Comparison of predicted protein secondary structures before and after SNP mutations. **(A)** Changes in protein secondary structures before and after mutation. **(B)** Secondary structure prediction before mutation. **(C)** Secondary structure prediction of after the SNP1 mutation. **(D)** Secondary structure prediction of after the SNP2 mutation. **(E)** Secondary structure prediction of after the SNP3 mutation. Solid red arrows indicate positions where protein secondary structures were predicted to increase after mutation, whereas hollow red arrows indicate positions where protein secondary structures were predicted to be absent after mutation.

**Table 4:** Predicted secondary structures of the *CCSER1* protein before and after single nucleotide polymorphism mutations.

SNPs	α-helix	Extended strand	β-turn	Random coil
Wild type	10.01% (90)	2.56% (23)	0.00% (0)	87.43% (786)
SNP1	9.57% (86)	1.78% (16)	0.00% (0)	88.65% (797)
SNP2	10.68% (96)	1.78% (16)	0.00% (0)	87.54% (787)
SNP3	10.01% (90)	2.00% (18)	0.00% (0)	87.99% (791)

Comparison of the predicted tertiary structures before and after mutation showed that the three mutations had relatively minor effects on the overall tertiary structure of the *CCSER1* protein. However, local structural changes were observed at the mutation sites. Specifically, SNP1 changed the side chain of the corresponding amino acid residue from an originally linear-like structure to a ring-like structure; SNP2 led to substitution of the local amino acid side-chain group; and SNP3 changed the side chain from an originally non-cyclic structure to a cyclic structure (Figure 3A). The predicted models showed minor local differences in side-chain conformation around the substituted residues, whereas the overall predicted protein structure remained largely similar.

**Figure 3:**
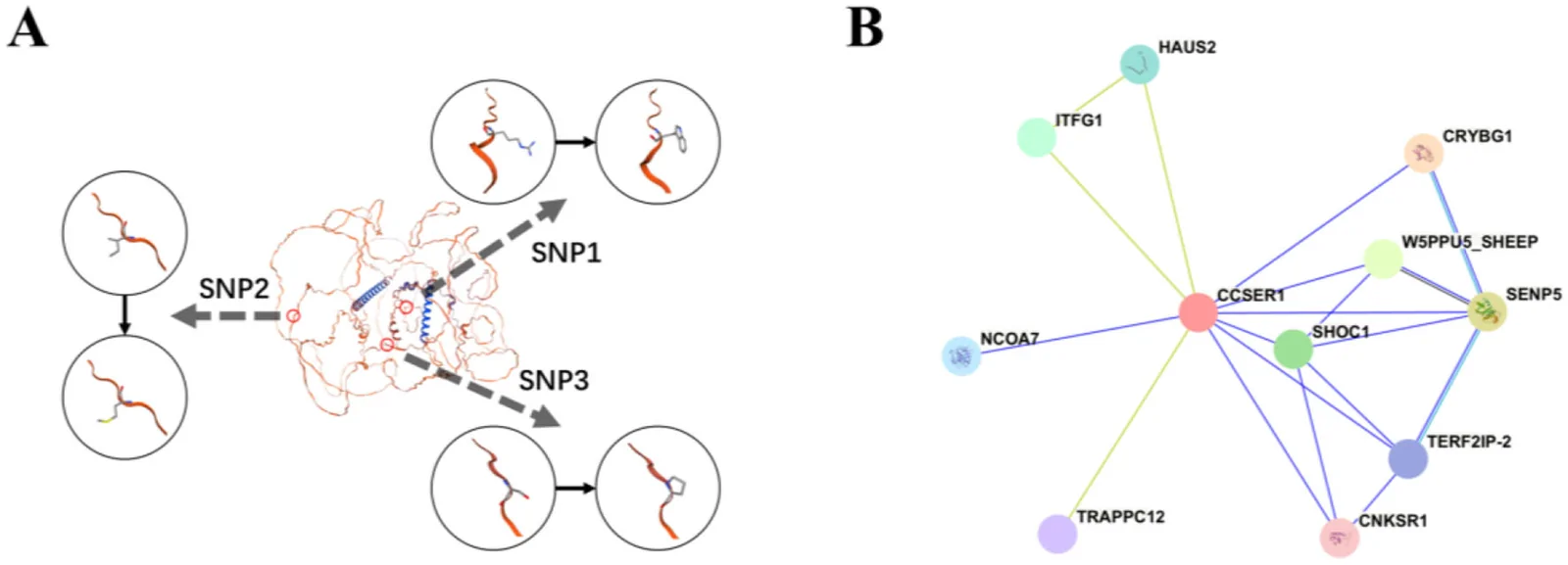
Comparison of predicted protein tertiary structures before and after SNP mutations and protein-protein interaction network of *CCSER1*. **(A)** Predicted three-dimensional structural model of the *CCSER1* protein. The central 3D model shows the overall spatial folding of the protein, and red circles indicate the spatial positions of the three identified missense mutations. The enlarged circular panels surrounding the model show the local side-chain conformational changes caused by the amino acid substitutions corresponding to SNP1, SNP2, and SNP3. **(B)** Potential protein-protein interaction network centered on *CCSER1* constructed using the STRING database under the low-confidence threshold. Nodes represent predicted interacting proteins. Dark blue edges indicate gene co-occurrence evidence, light blue edges indicate known interactions from curated databases, and yellow-green edges indicate potential associations based on text-mining evidence.

Furthermore, to explore the potential interacting molecules and regulatory network of *CCSER1* in biological processes, protein-protein interaction (PPI) analysis was performed using the STRING database. The results showed that, under the low-confidence score threshold, *CCSER1* was connected with several candidate proteins, including *SHOC1, CRYBG1* and *CNKSR1* (Figure 3B). Analysis of the network edge evidence indicated that the associations between *CCSER1* and the above proteins were mainly supported by gene co-occurrence and text-mining evidence, whereas strong evidence for direct physical complex interactions was not detected.

### Population genetic analysis of SNP loci in the *CCSER1* gene

Based on the genotyping data, population genetic analysis of the SNP loci in the *CCSER1* gene was further performed (Table 5). The results showed that three genotypes, GG, GA, and AA, were detected at SNP1, among which GG and GA were the predominant genotypes, and the G allele was the predominant allele. For SNP2, two genotypes, TT and TC, were detected, with the TT genotype and T allele being the predominant genotype and allele, respectively. For SNP3, three genotypes, AA, AG, and GG, were identified, among which the AG genotype and A allele were predominant.

**Table 5:** Population genetic analysis of the three SNP mutation loci in the *CCSER1* gene.

SNPs	Call rate	Genotype	Number	Freq	MAF	Ho	He	Ne	PIC	HWpval
SNP1	262 (98.9%)	GG (0.454) GA (0.485) AA (0.061)	119 127 16	G (0.697) A (0.303)	0.303	0.485	0.423	1.732	0.333	*P* = 0.02
SNP2	265 (100%)	TT (0.917) TC (0.083)	243 22	T (0.958) C (0.042)	0.042	0.083	0.081	1.087	0.076	*P* >0.05
SNP3	263 (99.2%)	AA (0.255) AG (0.532) GG (0.213)	67 140 56	A (0.521) G (0.479)	0.479	0.532	0.499	1.997	0.375	*P* >0.05

PIC ≥ 0.5 is high polymorphism, 0.25 ≤ PIC < 0.5 is moderate polymorphism, PIC < 0.25 is low polymorphism.

The observed heterozygosity (Ho) of all three loci was higher than the expected heterozygosity (He). Among them, SNP1 and SNP3 showed moderate polymorphism in the experimental population (0.25 ≤ PIC ≤ 0.5), although SNP1 deviated from Hardy–Weinberg equilibrium (HWE; *P* < 0.05). SNP2 showed low polymorphism (PIC < 0.25), with a minor allele frequency (MAF) lower than 0.05. However, because SNP2 was a missense mutation and conformed to HWE (*P* > 0.05), this locus was retained for subsequent phenotype association analysis.

### Descriptive statistics of phenotypic traits

Following phenotypic quality control, 287 litter-size (LS) records and 283 average birth weight per litter (ABW) records were retained for the reproductive traits, whereas the numbers of valid records for BW, WW, YW, ADG1, ADG2, and ADG3 were 234, 212, 209, 199, 204, and 205, respectively (Figure 4; Table 6). The numbers of individuals and record count for each genotype of the three SNPs are summarized in Supplementary Table S1. Mean LS was 1.93 ± 0.80 lambs per parity, with litter sizes of two lambs being the most frequent (44.2%), followed by one (32.7%), three (20.4%), and four lambs (2.7%). Mean ABW was 3.17 ± 0.62 kg, and 44.1% of the records were distributed within the 3.0–3.5 kg interval, followed by 23.4% within the 2.5–3.0 kg interval. For growth traits, mean BW, WW, and YW were 3.35 ± 0.75, 28.41 ± 6.87, and 53.71 ± 11.27 kg, respectively. The corresponding mean values for ADG1, ADG2, and ADG3 were 278.04 ± 72.06, 136.65 ± 32.77, and 92.14 ± 36.53 g/d, respectively. These phenotypic records were within the expected range for production performance and provided a data basis for subsequent phenotype association analysis.

**Figure 4:**
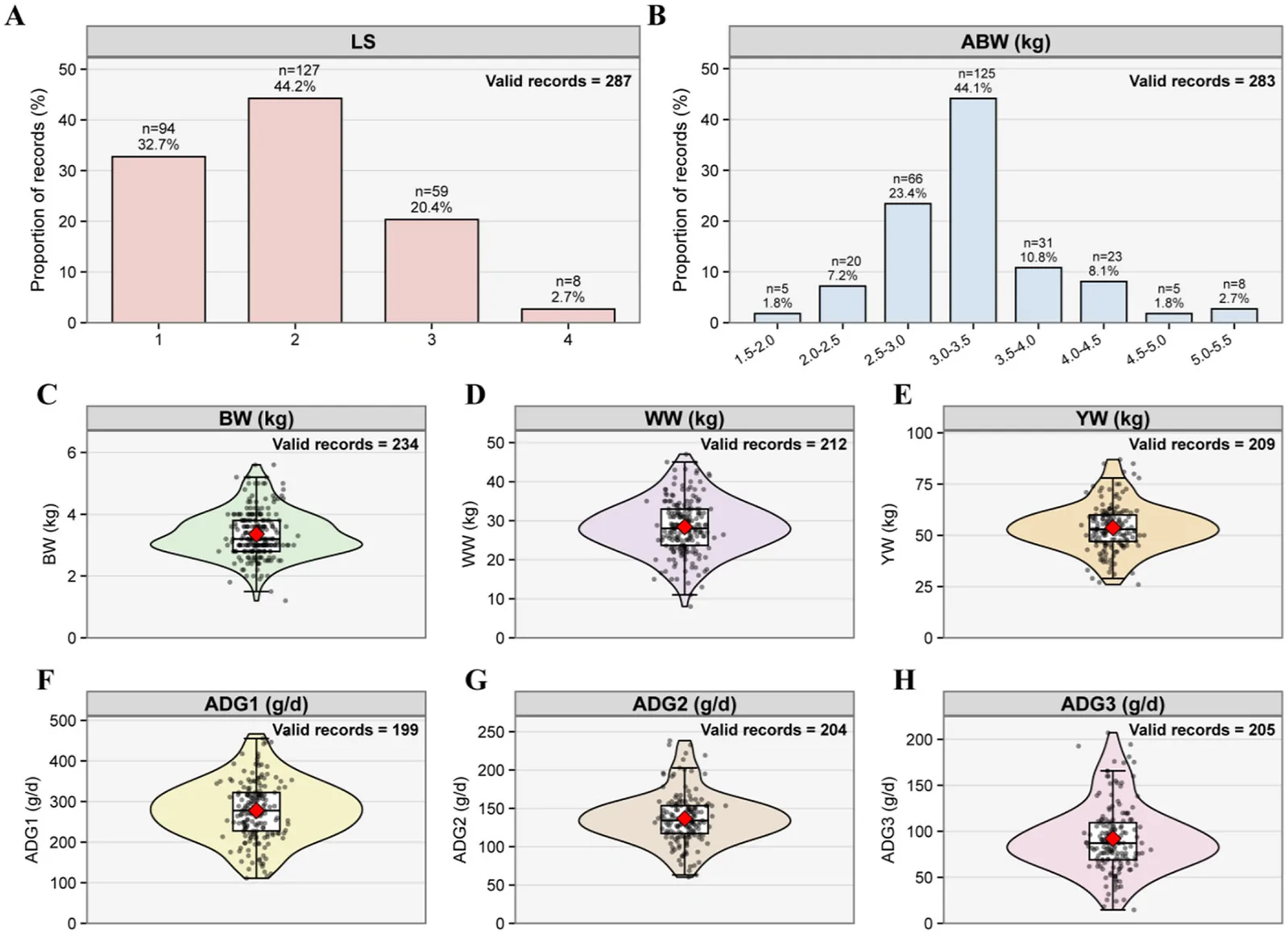
Distribution of eight phenotypic traits in the experimental population. **(A)** Frequency distribution of parity-specific litter size (LS), expressed as the proportion of valid lambing records for each litter-size category. **(B)** Frequency distribution of average birth weight per litter (ABW), grouped into 0.5-kg intervals. Numbers and percentages above the bars indicate the number and proportion of records within each category. **(C–H)** Violin plots showing the distributions of birth weight (BW), weaning weight (WW), yearling weight (YW), average daily gain from birth to weaning (ADG1), average daily gain from birth to 12 months of age (ADG2), and average daily gain from weaning to 12 months of age (ADG3), respectively. Individual dots represent phenotypic records, internal boxplots indicate the median and interquartile range, and red diamonds denote the arithmetic mean. The number of valid records used for each trait is indicated in the upper-right corner of each panel.

**Table 6:** Descriptive statistics of eight phenotypic traits in the experimental population.

Traits	Number of individuals	Record count	Mean	Min	Max	SD
LS	188	287	1.93	1	4	0.80
ABW(kg)	186	283	3.17	1.70	5.40	0.62
BW(kg)	234	234	3.35	1.20	5.60	0.75
WW(kg)	212	212	28.41	8.00	47.00	6.87
YW(kg)	209	209	53.71	26.00	87.00	11.27
ADG1(g/d)	199	199	278.04	111.11	466.67	72.06
ADG2(g/d)	204	204	136.65	60.27	238.36	32.77
ADG3(g/d)	205	205	92.14	14.55	207.27	36.53

Number of individuals denotes the number of individual animals contributing phenotypic data for each trait, whereas Record count denotes the total number of valid phenotypic observations included in the descriptive analysis. For repeatedly measured lambing traits (LS and ABW), multiple records could be obtained from the same ewe across different parities; therefore, the record count may exceed the number of sheep. For growth traits, each animal contributed a single valid observation, and thus the number of sheep was equal to the record count. Mean, Min, Max, and SD represent the arithmetic mean, minimum, maximum, and standard deviation, respectively.

### Association analysis between SNP loci in the *CCSER1* gene and phenotypic traits

JMP Pro software (version 18.0.0) was used to analyze the associations between the three *CCSER1* SNP loci and eight phenotypic traits. A single-stage mixed model was applied to evaluate the effects of individual SNP genotypes on phenotypic variation, followed by Bonferroni correction for multiple testing within each trait. The results after Bonferroni correction showed that SNP2 was significantly associated with ABW (adjusted *P* = 0.0024), SNP3 with YW (adjusted *P* = 0.022) and ADG2 (adjusted *P* = 0.039; Figure 5A; Table 7).

**Figure 5:**
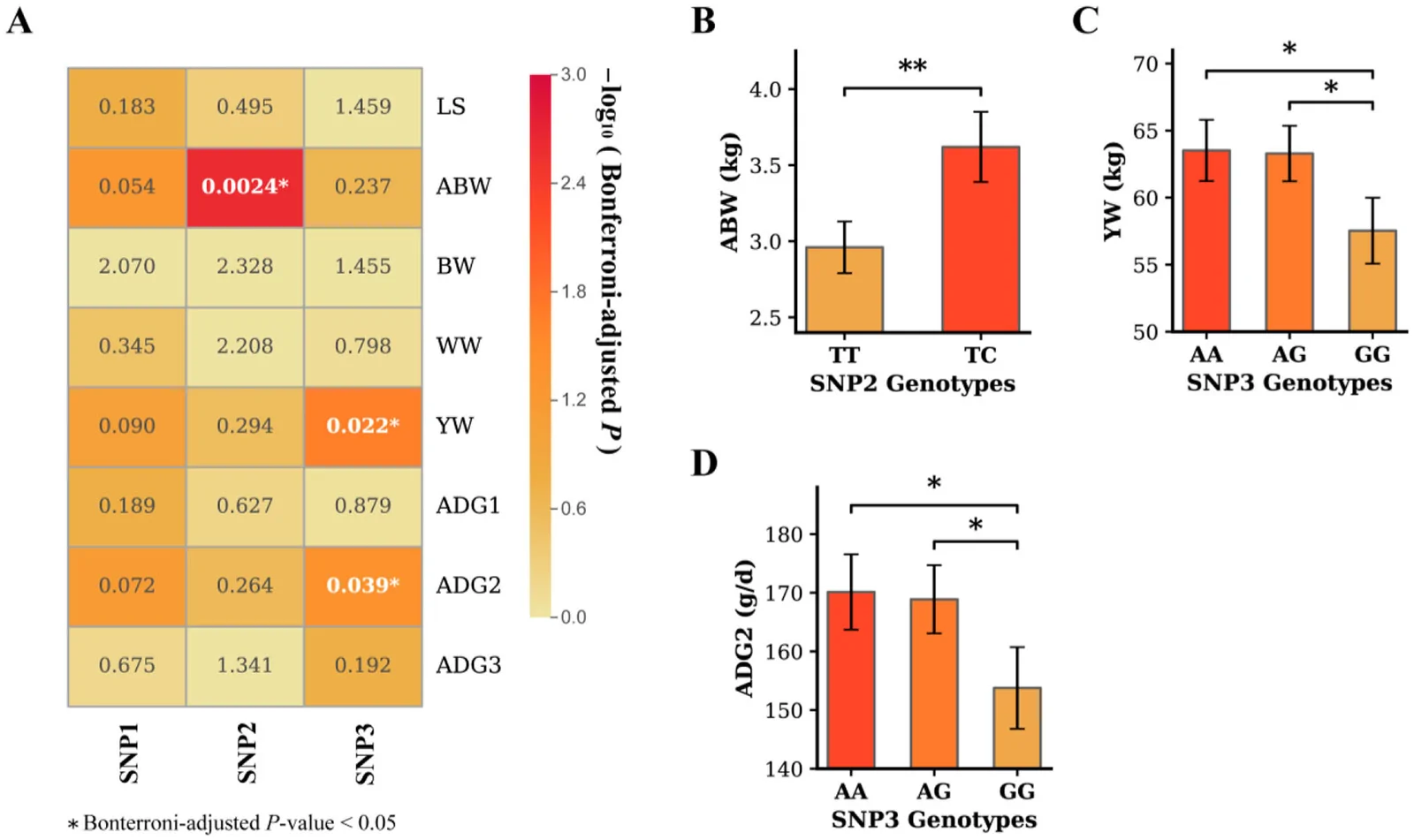
Association analysis between the three *CCSER1* SNP loci and phenotypic traits. **(A)** Heatmap showing Bonferroni-adjusted *P* values from the 24 SNP–trait association testsinvolving three *CCSER1* SNP loci and eight phenotypic traits. Cell colors represent −(Bonferroni-adjusted P values), and the corresponding adjusted P values are displayed within each cell. Asterisks indicate associations that remained statistically significant after Bonferroni correction (adjusted *P* < 0.05). **(B–D)** Genotype effects for traits showing significant associations after Bonferroni correction. Data are presented as adjusted least-squares means ± standard errors (SE). **(B)** Effect of SNP2 genotypes (TT and TC) on ABW; the SNP2–ABW association was significant after Bonferroni correction (adjusted *P* = 0.0024). **(C)** Effect of SNP3 genotypes (AA, AG, and GG) on YW; the SNP3–YW association remained significant after Bonferroni correction (adjusted *P* = 0.022). **(D)** Effect of SNP3 genotypes (AA, AG, and GG) on ADG2; the SNP3–ADG2 association remained significant after Bonferroni correction (adjusted *P* = 0.039). Pairwise comparisons among genotypes were performed using Tukey's HSD test following significant overall genotype effects. Horizontal lines indicate significant pairwise differences; ^*^ and ^**^ denote Tukey-adjusted comparisons with *P* < 0.05 and *P* < 0.01, respectively.

**Table 7:** Association analysis between SNP loci in the *CCSER1* gene and phenotypic traits.

SNPs	Type	LS	ABW (kg)	BW (kg)	WW (kg)	YW (kg)	ADG1 (g/d)	ADG2 (g/d)	ADG3(g/d)
SNP1	GG	2.21 ± 0.29	2.94 ± 0.20	3.04 ± 0.16	29.24 ± 1.72	61.74 ± 2.17	291.39 ± 19.03	164.33 ± 6.03	126.53 ± 6.62
	GA	2.35 ± 0.26	3.01 ± 0.18	3.00 ± 0.16	30.83 ± 1.67	65.17 ± 2.25	311.41 ± 18.56	174.66 ± 6.26	133.00 ± 6.65
	AA	1.56 ± 0.38	3.70 ± 0.27	2.86 ± 0.22	26.76 ± 2.48	58.24 ± 3.63	260.10 ± 28.22	156.70 ± 10.25	118.54 ± 10.83
Bonferroni-adjusted *P-value*		0.183	0.054	2.070	0.345	0.090	0.189	0.072	0.675
SNP2	TT	2.27 ± 0.26	2.96 ± 0.17^B^	2.99 ± 0.14	29.71 ± 1.59	62.21 ± 2.00	298.09 ± 17.67	166.34 ± 5.61	127.53 ± 5.95
	TC	1.90 ± 0.34	3.62 ± 0.23^A^	2.94 ± 0.22	30.35 ± 2.50	67.10 ± 3.53	324.73 ± 27.84	180.30 ± 9.84	134.20 ± 10.53
Bonferroni-adjusted *P-value*		0.495	0.0024	2.328	2.208	0.294	0.627	0.264	1.341
SNP3	AA	2.17 ± 0.26	3.13 ± 0.19	2.91 ± 0.17	29.74 ± 1.84	63.52 ± 2.28^a^	302.96 ± 20.53	170.13 ± 6.42^a^	130.23 ± 6.80
	AG	2.36 ± 0.28	3.09 ± 0.19	2.99 ± 0.15	29.97 ± 1.63	63.29 ± 2.07^a^	299.69 ± 18.23	168.88 ± 5.82^a^	129.55 ± 6.21
	GG	2.15 ± 0.36	2.70 ± 0.24	3.08 ± 0.18	27.75 ± 1.97	57.53 ± 2.46^b^	277.80 ± 21.89	153.77 ± 6.96^b^	116.52 ± 7.53
Bonferroni-adjusted *P-value*		1.459	0.237	1.455	0.798	0.022	0.879	0.039	0.192

Values are presented as adjusted least-squares means ± standard errors (SE). *P-values* represent the overall genotype effects obtained from the single-stage models. To account for multiple testing, Bonferroni correction was applied separately within each phenotypic trait. Associations with Bonferroni -adjusted *P-values* < 0.05 were considered statistically significant. For associations remaining significant after Bonferroni correction, pairwise comparisons among genotypes were performed using Tukey's HSD test. Different lowercase superscript letters indicate significant differences among genotypes (*P* < 0.05), whereas different uppercase superscript letters indicate highly significant differences among genotypes (*P* < 0.01).

Pairwise comparisons among genotypes were further performed using Tukey's HSD test for associations that remained significant after Bonferroni correction. At the SNP2 locus, individuals with the TC genotype exhibited higher ABW than those with the TT genotype (Figure 5B). At the SNP3 locus, individuals with the AA and AG genotypes showed higher YW and ADG2 values than individuals with the GG genotype (Figures 5C, D). These results indicate that SNP2 and SNP3 are associated with variation in growth-related traits, with specific genotypes showing higher phenotypic values for ABW, YW, and ADG2.

### LD analysis, haplotype reconstruction, and diplotype association analysis

Based on the LD analysis, SNP2 and SNP3 were located within Block 1, indicating linkage between the two loci (Figure 6A). Phasing of SNP2 and SNP3 in 263 individuals identified four chromosome-level haplotypes: T-A, T-G, C-A, and C-G. Their chromosome counts were 253, 251, 21, and 1, corresponding to frequencies of 48.10%, 47.72%, 3.99%, and 0.19%, respectively (Figures 6B, C). Pairing the two phased haplotypes carried by each individual yielded 6 diplotypes: T-A/T-G (*n* = 128, 48.67%), T-A/T-A (*n* = 57, 21.67%), T-G/T-G (*n* = 56, 21.29%), C-A/T-G (*n* = 11, 4.18%), C-A/T-A (*n* = 10, 3.80%), and C-G/T-A (*n* = 1, 0.38%) (Figures 6D). The 3 diplotypes with frequencies ≥5% were retained for phenotype association analyses. The numbers of individuals and record count for each diplotype are summarized in Supplementary Table S2.

**Figure 6:**
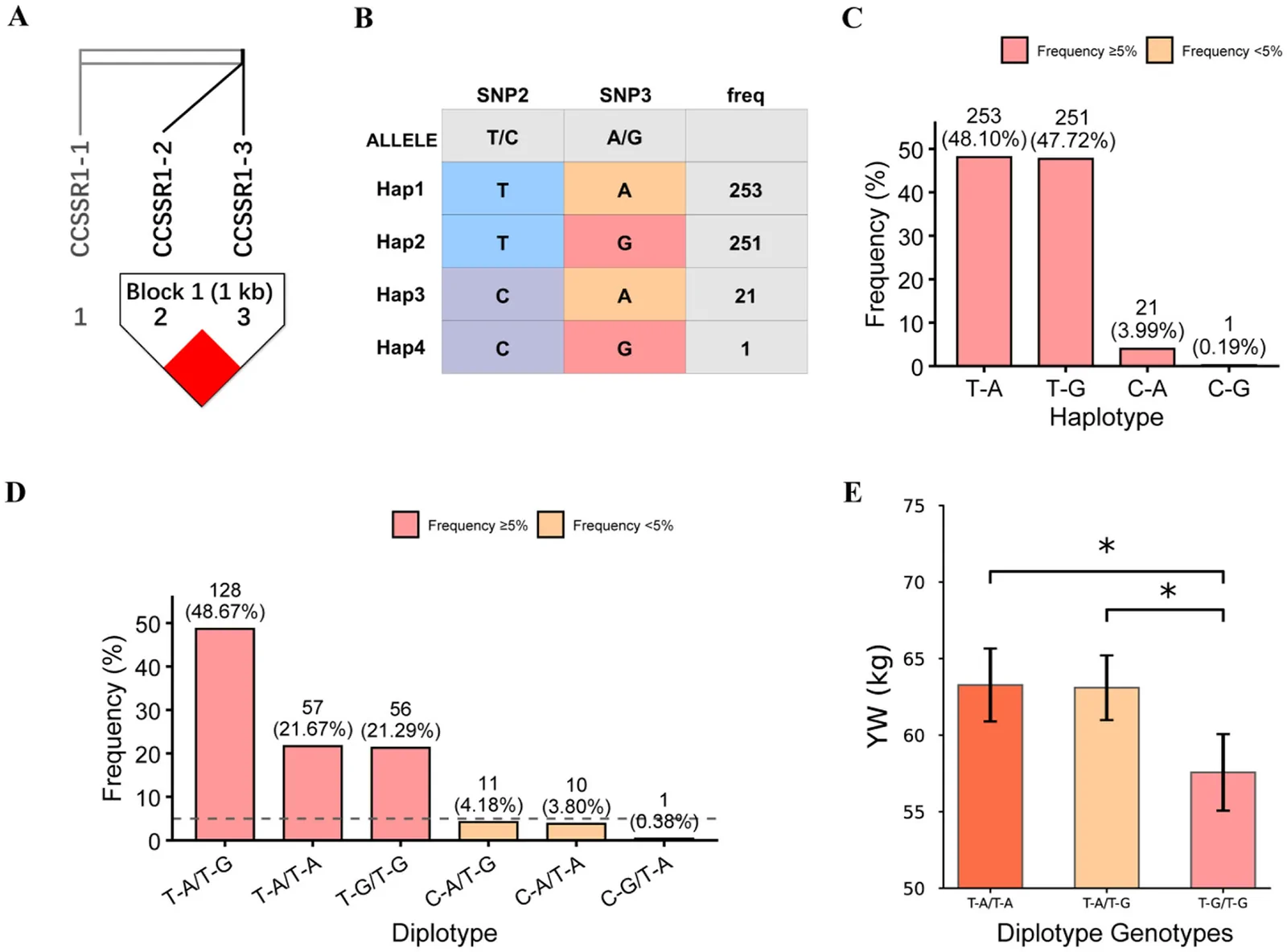
Linkage disequilibrium structure, haplotype and diplotype distribution of the *CCSER1* SNP2–SNP3 region and their association with YW. **(A)** Linkage disequilibrium analysis among the three *CCSER1* SNP loci. SNP2 and SNP3 formed a haplotype block. **(B)** Reconstruction of chromosome-level haplotypes based on the phased genotypes of SNP2 and SNP3. Four haplotypes (T-A, T-G, C-A, and C-G) were identified, and their frequencies were calculated from 526 phased chromosomes derived from 263 individuals. **(C)** Frequency distribution of the four chromosome-level haplotypes. Haplotypes with frequencies ≥5% and < 5% are indicated by different colors. **(D)** Frequency distribution of the six corresponding SNP2–SNP3 diplotypes in 263 individuals. The dashed horizontal line represents the 5% frequency threshold used for diplotype–phenotype association analyses. Diplotypes with frequencies below 5% were excluded from subsequent association testing. **(E)** Association analysis between SNP2–SNP3 diplotypes and YW. Values are presented as adjusted least-squares means ± standard errors (SE) The overall diplotype effects were evaluated using the single-stage mixed model, and Bonferroni correction was applied for multiple testing. For associations remaining significant after Bonferroni correction, pairwise comparisons among diplotypes were performed using Tukey's HSD test; ^*^ denote Tukey-adjusted comparisons with *P* < 0.05.

Diplotype-based association analysis after Bonferroni correction revealed significant effects of SNP2–SNP3 diplotypes on YW (adjusted *P* = 0.039). Subsequent pairwise comparisons using Tukey's HSD test indicated that individuals carrying the T-G/T-G diplotype exhibited significantly lower YW compared with those carrying T-A/T-A or T-A/T-G diplotypes, whereas no significant difference was observed between T-A/T-A and T-A/T-G diplotypes. (Figure 6E; Table 8).

**Table 8:** Association of *CCSER1* SNP2–SNP3 diplotypes with phenotypic traits in Junken meat sheep.

Diplotype	LS	ABW(kg)	BW(kg)	WW(kg)	YW(kg)	ADG1(g/d)	ADG2(g/d)	ADG3(g/d)
T-A/T-A	2.35 ± 0.28	2.91 ± 0.20	2.96 ± 0.17	30.10 ± 1.87	63.28 ± 2.38a	299.16 ± 21.37	169.59 ± 6.64	129.75 ± 7.07
T-A/T-G	2.42 ± 0.29	3.01 ± 0.19	3.01 ± 0.15	30.11 ± 1.62	63.10 ± 2.11a	300.85 ± 18.67	168.41 ± 5.89	129.38 ± 6.30
T-G/T-G	2.21 ± 0.36	2.65 ± 0.24	3.12 ± 0.18	27.81 ± 1.96	57.57 ± 2.50b	278.75 ± 22.42	154.02 ± 7.04	116.71 ± 7.61
Bonferroni-adjusted *P-value*	2.082	0.288	1.626	0.666	0.039	1.086	0.063	0.249

Values are presented as adjusted least-squares means ± standard errors (SE). *P-values* represent the overall genotype effects obtained from the single-stage models. To account for multiple testing, Bonferroni correction was applied separately within each phenotypic trait. Associations with Bonferroni -adjusted *P-values* < 0.05 were considered statistically significant. For associations remaining significant after Bonferroni correction, pairwise comparisons among genotypes were performed using Tukey's HSD test. Different lowercase superscript letters indicate significant differences among genotypes (*P* < 0.05).

### Tissue-specific expression profile of the *CCSER1* gene

The relative expression levels of *CCSER1* in 14 tissues were determined using RT-qPCR. The results showed that *CCSER1* expression levels in the lung, abomasum, and ovary were highly significantly higher than those in the liver, kidney, skin, brain, and pituitary gland (*P* < 0.01). Among all detected tissues, *CCSER1* showed the highest relative expression level in the abomasum, which was highly significantly higher than those in the lung and ovary (*P* < 0.01). No significant difference in *CCSER1* expression was observed between the lung and ovary (*P* > 0.05; Figure 7). In addition, the expression levels of *CCSER1* in the heart, skeletal muscle, rumen, duodenum, ileum, and uterus were below the limit of detection. These results provide a preliminary descriptive profile of the tissue distribution of *CCSER1* expression in Junken meat sheep.

**Figure 7:**
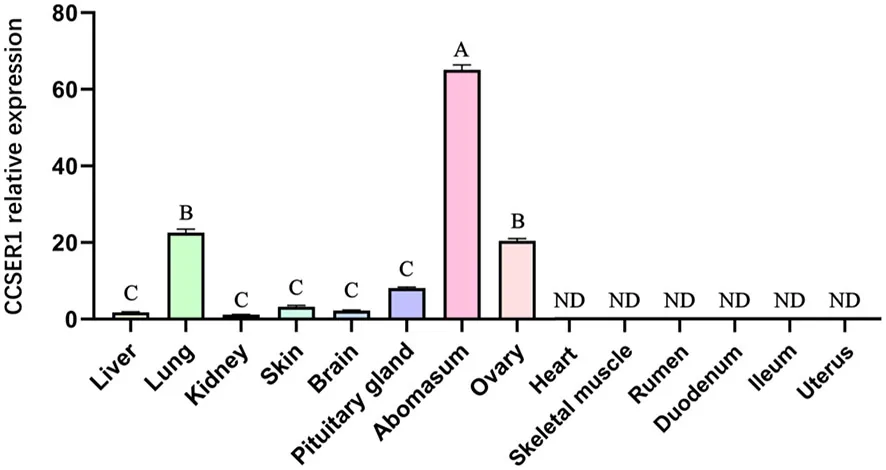
Relative expression levels of *CCSER1* in 14 tissues of the experimental individuals. Different uppercase letters indicate extremely significant differences among tissues (*P* < 0.01), whereas the same uppercase letter indicates no significant difference among tissues (*P* > 0.05). ND indicates that the expression level was below the limit of detection.

## Discussion

To date, extensive research has focused on molecular markers associated with growth and lambing traits in sheep. Previous investigations employing selection signature analysis, quantitative trait locus (QTL) mapping, and genome-wide association studies (GWAS) have yielded valuable evidence for screening candidate genes and advancing molecular breeding targeting reproductive and growth traits in sheep (18–21). However, sheep breeds vary greatly in genetic background, linkage disequilibrium patterns, allele frequencies and long-term selection objectives. Therefore, genome-wide candidate regions and genes cannot be directly utilized as molecular markers for marker-assisted selection within a single breed, necessitating further validation in the target experimental population (22, 23). Based on *CCSER1*, a candidate gene for litter size and yearling weight identified in our previous selection signature analysis, the present study identified polymorphisms in this gene in the Junken meat sheep population and systematically analyzed the associations of its genotypes and diplotypes with growth and lambing traits.

*CCSER1* encodes a protein containing coiled-coil domains and serine-rich regions and is mainly involved in biological processes such as cytoskeletal organization, cell migration, and cell division (7, 8). In recent years, the genetic regulatory potential and pleiotropic effects of *CCSER1* on economically important traits in livestock have gradually been revealed. Previous studies have shown that SNP variation in this gene significantly affects milk quality traits in Gannan yaks (24), that its copy number variation is associated with growth traits such as body weight and chest circumference in Chinese goat populations (25), and that *CCSER1* has been identified as an important candidate gene in a GWAS of growth traits in Red Angus cattle (26). Notably, Lukonina et al. (27) detected strong positive selection signatures in the genomic region harboring *CCSER1* in several sheep populations, including Salsk sheep, Suffolk sheep, and Texel sheep, using three within-population selection signature methods: integrated haplotype score (iHS), number of segregating sites by length (nSL), and integrated haplotype homozygosity pooled (iHH12). Collectively, these findings suggest that *CCSER1* may be involved in the genetic regulation of reproductive performance and growth rate in sheep. However, its genetic variation and phenotypic effects in Junken meat sheep remain to be further validated.

In this study, three missense SNPs in the *CCSER1* gene were identified using a candidate gene approach, including SNP1 ((XM_042251242.2: c.2557C>T) in exon 11 and SNP2 ((XM_042251242.2: c.1206A>G) and SNP3 ((XM_042251242.2: c.16T>C) in exon 2. Exploratory prediction analysis using SOPMA and SWISS-MODEL showed that the amino acid substitutions corresponding to these three SNPs may decrease the proportion of extended strand and increase the proportion of random coil, accompanied by structural changes in amino acid side chains. Similar missense mutation-induced shifts in protein secondary structure toward a more disordered and flexible state have been reported in studies of structural polymorphisms in breeds such as Subo Merino sheep, and such changes have been considered an important mechanism underlying alterations in conformational stability and substrate-binding affinity of target proteins (28). In addition, Schaefer and Rost reported, based on machine learning analyses, that side-chain alterations caused by single amino acid substitutions may change local protein conformation and thereby affect the structure and function of the encoded protein, as well as its physical binding capacity with cofactors (29, 30).

We further conducted population genetic analysis of the three SNP loci in Junken meat sheep. Junken meat sheep is a newly developed meat sheep breed independently bred in Xinjiang, China, in 2025, and has undergone long-term directional selection to improve lambing performance and growth performance (6). Population genetic parameters showed that SNP1 and SNP3 exhibited moderate polymorphism, whereas SNP2 showed low polymorphism. SNP2 and SNP3 conformed to Hardy-Weinberg equilibrium (HWE), while SNP1 deviated from HWE. Previous studies have demonstrated that deviations from Hardy–Weinberg equilibrium (HWE) at specific loci in livestock populations may arise from multiple biological and demographic factors, including long-term artificial selection, non-random mating, family structure, reduced effective population size, and population subdivision (31, 32). In livestock breeding populations, intensive selection programs and unequal reproductive contributions of elite individuals can reshape allele-frequency distributions and genetic relationships among individuals, thereby influencing genotype equilibrium at particular loci (33). Therefore, the deviation of SNP1 from HWE observed in the Junken meat sheep population may reflect the combined effects of breeding history, selection practices, and population genetic characteristics. Considering that the association signals of SNP1 did not remain significant after Bonferroni correction, SNP1 was not regarded as a reliable molecular genetic marker for growth or lambing-related traits in the current population. Interestingly, although SNP2 showed low polymorphism and a minor allele frequency (MAF) lower than 0.05 in the present population, individuals with the heterozygous genotype had highly significantly higher ABW than those with the wild-type genotype. Manolio et al. (34) reported that some low-frequency variants with significant phenotypic effects are important for explaining complex quantitative traits. In livestock populations, the presence of low-frequency favorable alleles may be associated with recent mutation, introgression, genetic drift, population-specific selection, or an ongoing breeding process in which such alleles have not yet been sufficiently fixed (35). Therefore, SNP2 and SNP3was retained in this study as potentially favorable variants and included in subsequent diplotype and phenotype association analyses to further evaluate its genetic effect and potential breeding value.

To further evaluate the robustness of the associations between *CCSER1* variants and phenotypic traits in Junken meat sheep, genotype–phenotype and diplotype–phenotype associations were systematically assessed, followed by Bonferroni correction for multiple testing. Different mutation loci in *CCSER1* showed significant effects on growth and lambing-related traits. Specifically, the TC genotype at SNP2 were identified as favorable genotypes for ABW, whereas the AA and AG genotypes at SNP3 showed significant positive effects on YW and ADG2. ABW is a key indicator reflecting maternal nutrient allocation during gestation and fetal developmental potential, and it may further affect postnatal health status, growth and development, and subsequent production performance of lambs (36). Meanwhile, body weight at 12 months old and average daily gain from birth to 12 months old are important reference indicators for evaluating the economic performance of commercial meat sheep (37). Therefore, the results of this study suggest that *CCSER1* variants may be more closely involved in biological processes related to fetal developmental quality and postnatal growth and development.

Notably, no significant differences in BW were observed among individuals with different genotypes, whereas significant phenotypic divergence was observed in YW and ADG2. Khazaei-Koohpar et al. (38) reported that, during the early postnatal stage in sheep, maternal genetic effects and maternal environmental effects have additive effects on lamb body weight. After weaning, as lambs enter the stage of independent feeding, individual additive genetic effects gradually increase, leading to more pronounced differentiation in growth phenotypes. This finding not only suggests that *CCSER1* may be involved in mid- to late-stage postweaning growth and development in sheep, but is also consistent with the study by Li et al. (11), who identified *CCSER1* as a growth-related candidate gene in a GWAS of body weight traits at different ages in Hu sheep. To evaluate the cumulative genetic effects generated by multiple loci, haplotype analysis was performed based on the coordinated inheritance of linked variants within the gene (39). To evaluate the joint inheritance pattern of SNP2 and SNP3, chromosome-level haplotypes and individual diplotypes were reconstructed from phased genotype data. The two major haplotypes, T-A and T-G, together accounted for more than 95% of phased chromosomes, whereas C-containing haplotypes occurred at low frequencies. Among the six reconstructed diplotypes, T-A/T-G, T-A/T-A, and T-G/T-G were the three common classes retained for association analysis. The results showed that individuals carrying T-A/T-A and T-A/T-G showed significantly higher YW than those carrying T-G/T-G. This diplotype evidence not only helps overcome the limitation of weak effects from individual SNPs but also further supports the potential breeding value of *CCSER1* as a molecular marker for improving meat sheep performance.

To further explore the potential biological basis underlying the association between *CCSER1* and phenotypic differences in Junken meat sheep, a tissue expression profile analysis was performed in this study. The results showed that *CCSER1* was predominantly expressed in the abomasum, ovary, and lung, with the highest expression level observed in the abomasum. The abomasum is the true stomach of ruminants and is the only one of the four gastric compartments that retains the ancestral mammalian glandular secretory function and performs the core function of protein digestion. It secretes large amounts of hydrochloric acid and pepsin (40, 41). Therefore, the high expression of *CCSER1* in the abomasum may be associated with the maintenance of abomasal tissue function, epithelial cell renewal, or digestion-related physiological processes. Previous studies have reported that *CCSER1* may participate in processes such as cytoskeletal organization, cell migration, and cell division (8). Ramljak et al. (42) found that *CCSER1* was one of the genes under strong positive selection in local sheep breeds with high feed intake, suggesting a potential link between this gene and traits related to feed intake, digestion, or growth and development. The increased mechanical friction and feeding load caused by high feed intake may keep abomasal epithelial cells in a long-term state of shedding and renewal (43), accompanied by increased expression of many genes related to cell growth and proliferation in abomasal tissue (44).

Interestingly, *CCSER1* also showed a certain level of expression in the ovary. Combined with the significant positive effect of the SNP2 mutation on ABW in the association analysis, this finding suggests that *CCSER1* may have a potential relationship with ovarian function or early fetal developmental processes. ABW is jointly affected by multiple factors, including maternal nutrient supply, the gestational environment, placental function, and fetal developmental status (45, 46). Therefore, the association between *CCSER1* and ABW may reflect its potential involvement in reproductive tissues or physiological processes related to fetal development. This is biologically consistent with the recent finding by Wang et al. (47), who reported that MMP16 may improve lambing performance in breeds such as Lop sheep by regulating the microenvironment of follicular development.

In addition, complex reproductive and growth-related traits such as ABW, YW, and ADG2 are often difficult to fully explain solely by the local expression pattern of a single gene (48–50). Previous studies based on the omnigenic model and weighted gene co-expression network analysis (WGCNA) in livestock have suggested that complex animal traits are not determined only by isolated major-effect genes, but are also shaped by the coordinated and integrated regulation of numerous small-effect genes within large and highly interconnected regulatory networks (51–54). In the present study, PPI analysis indicated that *CCSER1* occupied a node within the interaction network and was connected with 10 proteins, including SHOC1, suggesting that *CCSER1* may participate in the coordinated regulation of complex production traits and serve as a candidate gene associated with these traits in Junken meat sheep. This potential indirect cooperative mechanism requires further investigation through functional validation.

## Conclusion

In conclusion, three missense SNPs, namely SNP1 (XM_042251242.2: c.2557C>T), SNP2 (XM_042251242.2: c.1206A>G), and SNP3 (XM_042251242.2: c.16T>C), were identified in the coding region of the *CCSER1* gene. After single-stage association analysis and correction for multiple testing, SNP2 and SNP3 were significantly associated with growth- and early development-related traits, including ABW, YW, and ADG2. In addition, sheep carrying the T-A/T-A or T-A/T-G diplotypes formed by SNP2 and SNP3 exhibited significantly higher YW than those carrying the T-G/T-G diplotype. The high expression of *CCSER1* in the abomasum and its moderate expression in the ovary further suggest that this gene may be involved in cellular renewal processes in ruminant digestive tissues and maternal reproductive organs. SNP2, SNP3, and the T-A/T-A and T-A/T-G diplotypes within the *CCSER1* gene may therefore represent promising candidate markers for marker-assisted selection of lambing- and growth-related traits in Junken meat sheep, potentially providing a basis for improving average birth weight per litter, yearling weight, and average daily gain.

## Data Availability

The data supporting the conclusions of this study are included in the article and Supplementary Material. The study did not generate high-throughput sequencing data; genotyping was limited to three predefined CCSER1 SNP loci using the Sequenom MassARRAY^®^ SNP assay. Individual-level phenotypic records are not publicly available because they are subject to institutional and third-party data-use restrictions associated with the breeding population. Requests for access to additional de-identified data should be directed to the corresponding authors and will be considered subject to the applicable institutional and data-owner permissions.
