# Supplementary material for: Identification of CCSER1 missense variants and diplotypes as molecular markers for growth and lambing-related traits in Junken meat sheep
Source: Front Vet Sci. 2026 Sep 17;13:1922937. doi: 10.3389/fvets.2026.1922937 (PMC13627002; doi:10.3389/fvets.2026.1922937)
Supplement: Supplementary file 1 [file Table_1.DOCX]

Table S1. Number of individuals and phenotypic records included in the single-SNP association analyses for each genotype group.

| Trait | SNP | Genotype | Number of individuals | Record count |
| --- | --- | --- | --- | --- |
| LS | SNP1 | GG | 66 | 99 |
|  |  | GA | 107 | 170 |
|  |  | AA | 15 | 18 |
|  | SNP2 | TT | 170 | 259 |
|  |  | TC | 18 | 28 |
|  | SNP3 | AA | 51 | 87 |
|  |  | AG | 114 | 170 |
|  |  | GG | 23 | 30 |
| ABW | SNP1 | GG | 66 | 99 |
|  |  | GA | 107 | 169 |
|  |  | AA | 13 | 15 |
|  | SNP2 | TT | 168 | 257 |
|  |  | TC | 18 | 26 |
|  | SNP3 | AA | 48 | 84 |
|  |  | AG | 115 | 168 |
|  |  | GG | 23 | 31 |
| BW | SNP1 | GG | 106 | 106 |
|  |  | GA | 111 | 111 |
|  |  | AA | 14 | 14 |
|  | SNP2 | TT | 214 | 214 |
|  |  | TC | 20 | 20 |
|  | SNP3 | AA | 59 | 59 |
|  |  | AG | 125 | 125 |
|  |  | GG | 49 | 49 |
| WW | SNP1 | GG | 98 | 98 |
|  |  | GA | 101 | 101 |
|  |  | AA | 13 | 13 |
|  | SNP2 | TT | 195 | 195 |
|  |  | TC | 17 | 17 |
|  | SNP3 | AA | 50 | 50 |
|  |  | AG | 118 | 118 |
|  |  | GG | 42 | 42 |
| YW | SNP1 | GG | 97 | 97 |
|  |  | GA | 99 | 99 |
|  |  | AA | 10 | 10 |
|  | SNP2 | TT | 194 | 194 |
|  |  | TC | 15 | 15 |
|  | SNP3 | AA | 51 | 51 |
|  |  | AG | 117 | 117 |
|  |  | GG | 38 | 38 |
| ADG1 | SNP1 | GG | 95 | 95 |
|  |  | GA | 92 | 92 |
|  |  | AA | 12 | 12 |
|  | SNP2 | TT | 183 | 183 |
|  |  | TC | 16 | 16 |
|  | SNP3 | AA | 46 | 46 |
|  |  | AG | 112 | 112 |
|  |  | GG | 40 | 40 |
| ADG2 | SNP1 | GG | 98 | 98 |
|  |  | GA | 93 | 93 |
|  |  | AA | 10 | 10 |
|  | SNP2 | TT | 189 | 189 |
|  |  | TC | 15 | 15 |
|  | SNP3 | AA | 51 | 51 |
|  |  | AG | 112 | 112 |
|  |  | GG | 38 | 38 |
| ADG3 | SNP1 | GG | 94 | 94 |
|  |  | GA | 98 | 98 |
|  |  | AA | 10 | 10 |
|  | SNP2 | TT | 191 | 191 |
|  |  | TC | 14 | 14 |
|  | SNP3 | AA | 48 | 48 |
|  |  | AG | 116 | 116 |
|  |  | GG | 37 | 37 |

**Note:** *Number of individuals* represents the number of unique animals with available phenotypic and genotypic information for each trait–SNP analysis. *Record count* represents the total number of phenotypic observations included in the corresponding statistical model. For repeatedly measured reproductive traits (e.g., LS and ABW), an individual ewe could contribute records from more than one parity; therefore, the number of records may exceed the number of individuals.

Table S2. Number of individuals and phenotypic records included in the SNP2–SNP3 diplotype association analyses for each diplotype group.

| Trait | Diplotype | Number of individuals | Record count |
| --- | --- | --- | --- |
| LS | T-A/T-A | 38 | 71 |
|  | T-A/T-G | 107 | 158 |
|  | T-G/T-G | 23 | 30 |
| ABW | T-A/T-A | 41 | 71 |
|  | T-A/T-G | 107 | 158 |
|  | T-G/T-G | 23 | 31 |
| BW | T-A/T-A | 50 | 50 |
|  | T-A/T-G | 114 | 114 |
|  | T-G/T-G | 49 | 49 |
| WW | T-A/T-A | 43 | 43 |
|  | T-A/T-G | 108 | 108 |
|  | T-G/T-G | 42 | 42 |
| YW | T-A/T-A | 44 | 44 |
|  | T-A/T-G | 111 | 111 |
|  | T-G/T-G | 39 | 39 |
| ADG1 | T-A/T-A | 40 | 40 |
|  | T-A/T-G | 102 | 102 |
|  | T-G/T-G | 40 | 40 |
| ADG2 | T-A/T-A | 44 | 44 |
|  | T-A/T-G | 105 | 105 |
|  | T-G/T-G | 39 | 39 |
| ADG3 | T-A/T-A | 41 | 41 |
|  | T-A/T-G | 111 | 111 |
|  | T-G/T-G | 38 | 38 |

**Note:** *Number of individuals* represents the number of unique animals with available phenotypic and diplotype information for each trait analysis. *Record count* represents the total number of phenotypic observations included in the corresponding statistical model. For repeatedly measured reproductive traits (e.g., LS and ABW), an individual ewe could contribute records from more than one parity; therefore, the number of records may exceed the number of individuals.
